# Worth of the Christian Ministry

**Published:** 1869-11

**Authors:** 


### HALL'S JOURNAL OF HEALTH.

OUR LEGITIMATE SCOPE IS ALMOST BOUNDLESS: FOR WHATEVER BEGETS PLEASURABLE AND HARMLESS FEELINGS, PROMOTES HEALTH; AND WHATEVER INDUCES DISAGREEABLE SENSATIONS, ENGENDERS DISEASE.

*We aim to show how Disease may be avoided, and that it is best, when sickness comes, to take no Medicine without consulting an educated Physician.*

VOL. XVI.] NOVEMBER, 1869. [No. 11.

### WORTH OF THE CHRISTIAN MINISTRY.

"He went about doing good, and had not a place where to lay his head," comprises the life of the author of our holy religion during his earthly pilgrimage; and such has been the actual experience of multitudes of the best ministers of the Gospel the world ever saw, down to the present day, and will be, until time shall be no longer. The reason is, "Even so, Father, for it seemeth good in thy sight." God wills it, and that is reason enough. It was not intended that their reward should be in this world, but that their works should follow. The salaries of the clergy of the United States do not average five hundred dollars a year, and yet, as a class, they are the best educated, the most influential, the most active, refined, and elevated of the nation. With less culture, with less character, with less mental power, there are men, all over the land, who earn from one to twenty-five thousand dollars a year. But look at the results. Taking them as they come, the biographies of a hundred clergymen who had families show that, of their sons, one hundred and ten became ministers; and of the remainder of the sons, by far the larger number rose to eminence as professional men, merchants, and scholars.

As to the daughters, their names are merged into others; but there is a significant fact, which we do not remember to have seen noticed in that connection, that not only here, but in England, where titles are so highly prized, and the possession of "gentle blood" is a passport to high places, it is very often referred to, as a matter of note, as indicating safety and respectability—"His mother was the daughter of a clergyman." We will venture the opinion, that three-fourths of the great men of this nation are not over two degrees removed from clergymen's families, or from families strictly religious. When it can be said of a man, or woman, that the father, or grandfather, was a clergyman, there is a feeling within us of a certain elevation of character, a kind of guarantee of respectability of blood, of purity and integrity.

We need not ask if the history of any other hundred families, taken as they come, of renowned generals, of great statesmen, of successful merchants, of splendid orators, or eminent physicians and lawyers, can give another hundred and ten sons to occupy positions as respectable as their own—never, nor is there any approach to it.

Half of our "successful" merchants die in poverty eventually, while their sons grow up in habits of idleness and early dissipation (as is also the case, more or less, with most of the children of prominent men); disease wastes their bodies, the disease which originates from demoralizing indulgences; while the mind itself, from the want of a sufficient stimulus to energy, dwindles to a point below mediocrity. As to the daughters of the worldly eminent, what becomes of them? They devote themselves to fashion, and dress, and revelry, and a vain show; to be wooed and won by men who grew up without occupation, looking for their father's fortune; or by adventurers, who live by their wits—the end being, that most incongruous of all combinations, poverty and pride, with that most bootless of all ambitions, to keep up appearances—than which, a more hideous, painful, and unsatisfying struggle, no human being could ever encounter. In short, the rarest of all rare things in this country is, to find a grandchild enjoying the fortune or position of the grandparents, if, indeed, there be any grandchild at all; for disappointment, fed by want of occupation, grinds out the life, and quite early, too, of the children of the world. A daughter of one of the richest men in America ten years ago, herself the wife of a great man, has an attendant, whose whole duty it is to keep her from intoxication. Another daughter drank ravenously her cologne water, for want of spirits or opium, and died in her infatuation. One of the most splendid women of our time, degrades herself, at varying intervals, by a regular drunken bout.

Thus it is, that we regard the privation and the poverty of the clergy as a means of perpetuating the mental vigor, the real thrift and position of our nation. They are literally the salt of the earth; not only its preserving principle of to-day, but for future time. Great reason, then, have clergymen, and clergymen's wives, to bear their present burdens of daily labor and daily stinting. Plain dwellings, plain clothing, plain food—and even that not over abundant—may be their portion here below; but, beside the reward above, they will be honored and affectionately remembered, when they are dead and gone, by the very people for whom they labored, and who allowed them to live on scanty salaries. But there is another and a higher reward than human appreciation—their influences for good are perpetuated in their children, bodily, mental, and moral, and this is the pith of this article. The straitened circumstances of ministers' families give that kind of practical teaching, that suitableness and preparation for practical life, in after years, which is so necessary to success. Having nothing to look for, but the results of their own exertions, they early learn to be self-reliant and thoughtful, impressing the whole character with a manly dignity, which everywhere commands respect. In addition, knowing they must depend on themselves, they at once begin those activities which ensure health, while, by the stern necessity of extremely plain fare and homely accommodations, with the impossibility of means to secure luxurious indulgences, or the opportunities of frivolous amusements and trifling recreations, their bodies grow up to a vigor and a healthfulness which give that power to mind which commands success in every department of human life. In addition to all these, there are those moral teachings, which fall as ceaselessly as the dews of the sky, and as gently, from earliest infancy, moulding the character, and fixing those principles of action, which so well sustain their possessors in life's conflicts, and which elevate all with whom they come in contact.

Take courage then, ye "ministers of the word." You may feel straitened, and at times greatly discouraged, "because of the way" through which you are called to pass; but look at the reward! The affectionate and respectful remembrance of those you once preached to, long after you are dead and gone, and forgotten it may be, by the great world, but never by them—just as you now think with reverential gratitude of the men who were your early ministers, and will continue to think thus, until life's latest hour! And then, what solid satisfaction is there, in leaving sons and daughters behind you who shall perpetuate your influences, and live out your principles for generations to come! That compulsory activity, and that compulsory plainness of living, and that dearth of amusement and recreation and "enjoyment," falsely so called, which your limited means entails on your children, these are the things that will make them what you would really have them to be—true men and women. They do not, it is true, inherit from you millions of money, but you entail on them that necessity of industrious activity, and that rational temperance, which are at once the foundation of human happiness and human success

